# A Robust, Non-Cooperative Localization Algorithm in the Presence of Outlier Measurements in Ocean Sensor Networks

**DOI:** 10.3390/s19122708

**Published:** 2019-06-16

**Authors:** Xiaojun Mei, Huafeng Wu, Jiangfeng Xian, Bowen Chen, Hao Zhang, Xia Liu

**Affiliations:** 1 Merchant Marine College, Shanghai Maritime University, Shanghai 201306, China; xjmei94@163.com (X.M.); xianjiangfeng0310@163.com (J.X.); bowenchen1995@gmail.com (B.C.); haozhangsmu@163.com (H.Z.); xialiu@shmtu.edu.cn (X.L.); 2 Department of Informatics, Linnaeus University, Växjö 351 06, Sweden

**Keywords:** bisection method, ocean sensor networks, outlier measurements, non-cooperative localization, received signal strength indication

## Abstract

As an important means of multidimensional observation on the sea, ocean sensor networks (OSNs) could meet the needs of comprehensive information observations in large-scale and multifactor marine environments. In what concerns OSNs, accurate location information is the basis of the data sets. However, because of the multipath effect—signal shadowing by waves and unintentional or malicious attacks—outlier measurements occur frequently and inevitably, which directly degrades the localization accuracy. Therefore, increasing localization accuracy in the presence of outlier measurements is a critical issue that needs to be urgently tackled in OSNs. In this case, this paper proposed a robust, non-cooperative localization algorithm (RNLA) using received signal strength indication (RSSI) in the presence of outlier measurements in OSNs. We firstly formulated the localization problem using a log-normal shadowing model integrated with a first order Taylor series. Nevertheless, the problem was infeasible to solve, especially in the presence of outlier measurements. Hence, we then converted the localization problem into the optimization problem using squared range and weighted least square (WLS), albeit in a nonconvex form. For the sake of an accurate solution, the problem was then transformed into a generalized trust region subproblem (GTRS) combined with robust functions. Although GTRS was still a nonconvex framework, the solution could be acquired by a bisection approach. To ensure global convergence, a block prox-linear (BPL) method was incorporated with the bisection approach. In addition, we conducted the Cramer–Rao low bound (CRLB) to evaluate RNLA. Simulations were carried out over variable parameters. Numerical results showed that RNLA outperformed the other algorithms under outlier measurements, notwithstanding that the time for RNLA computation was a little bit more than others in some conditions.

## 1. Introduction

Wireless sensor networks (WSNs) are self-organizing networks consisting of numerous sensor nodes connected by some means of communication. Because of their small size, low energy consumption, strong robustness, flexible layout, and rapid networking for nodes, WSNs are now widely used in a large number of fields [1,2,3,4]. One of the vital applications is to conduct ocean sensor networks (OSNs) to monitor ocean environments in the case of forecasting climate changes [5]. To fulfill this task, a tremendous number of sensor nodes, such as buoys that are sensitive to environmental factors, are deployed on the ocean surface. Considering the cost of nodes, only a few sensor nodes are armed with a global positioning system (GPS), called anchor nodes [6,7], while the others are unknown nodes. It should be noted that data collected by sensor nodes are meaningful only when the latter is properly geo-referenced [8]. Hence, the critical issue that needs to be tackled in OSNs is to acquire location information of the unknown nodes with assistance from the anchor nodes. 

Regarding localization in OSNs, non-cooperative localization (NCL) and cooperative localization (CL) schemes are considered the major scenarios [9]. In CL, the unknown nodes are able to communicate with the rest of the sensor nodes (anchor nodes and other unknown nodes) within the communication range, whereas they cannot in NCL. In general, the complexity, computational time, and energy consumption in CL are far more than that of NCL, albeit more accurate as well [10]. In order to achieve long-term monitoring of the ocean, the scheme with less energy consumption seems to be a better choice. In this case, we focus more on the NCL scheme in OSNs. Basically, NCL contains five measurement techniques: (1) time of arrival (TOA), (2) time difference of arrival (TDOA), (3) angle of arrival (AOA), (4) time of flight (TOF), and (5) received signal strength indication (RSSI) [8]. As for the localization accuracy, TOA, TDOA, AOA, and TOF may outperform RSSI at the cost of extra apparatuses in some circumstances. Besides, TOA, TDOA, and TOF have to synchronize time during localization. On the contrary, there is no need to arm extra apparatuses and synchronize time in RSSI. Therefore, RSSI is considered a cost-effective and energy-saving method widely used in OSNs [11]. In this case, we used RSSI as the measurement technique in this paper.

Past years have witnessed development of research on localization in OSNs. Considerable efforts have been investigated in recent years [12,13,14,15,16,17,18,19,20,21,22,23,24,25]. To mention a few, an optimal sensor placement strategy that depended on the required task at hand was proposed in [13]. This strategy could run in the presence of Gaussian noise in the range-based scheme. Then, the authors further proposed a multiple underwater target positioning method in [18] with the strategy exploited. The localization problem in [18] was transformed into an optimization problem. Two-phase localization was presented, where convex optimization tools were utilized for single target localization, and Pareto optimization tools were used when it came to multiple target localizations. Similar two-phase work was proposed in [23]. In the first phase, a particle swarm optimization (PSO)-based localization algorithm was employed to locate the unknown nodes. For the sake of avoiding the case that some of the unknown nodes had not been localized in the first phase, a circle-based, range-free localization method was presented in the second phase.

Nevertheless, two-phase localization methods function well only when the anchor nodes are not faulted. In this matter, to avoid anchor node failure, a fault-resilient localization method was proposed in [24]. The authors utilized multiple linear regression to learn mobility behaviors from the neighbors of unknown nodes. The authors in [12] presented a maximum likelihood estimator under Gaussian noise to deal with anchor node uncertainty. In addition, some anchor-free localization methods have been presented to handle anchor node failure localization. Li et al. proposed an anchor-free localization mechanism using belief propagation integrated with dead reckoning [25]. The authors in [16] presented a signal reflection-enabled, acoustic-based localization scheme (UREAL), which was entirely an anchor-free approach. This scheme established the refracted–surface–reflected link for all sensor nodes using RSSI information. Then, the authors utilized AOA ranging in the position estimation. In addition to localization accuracy, energy consumption is considered another limitation in localization. Guo et al. proposed an energy-aware localization method in [15], wherein the authors took ships as anchor nodes to engage in energy-saving. Yan et al. presented an autonomous underwater vehicle (AUV)-aided localization scheme, wherein active nodes and passive nodes were included [14]. Yan et al. proposed an energy-efficient localization algorithm in [17]. The algorithm converted the localization problem into a convex optimization problem using norm relaxation and semidefinite relaxation.

However, it should be emphasized that outlier measurements may occur frequently and inevitably because of the multipath effect, unintentional or malicious attack, and signal shadowing caused by waves [10,26,27]. To the best of our knowledge, only a few papers took outlier measurements into consideration when it came to localization in OSNs. In [28], the authors applied support vector data description (SVDD) to detect outlier measurements and then exploited auto-associative kernel regression (AAKR) to correct deviations. The authors in [29] employed half quadratic minimization to solve the localization problem in the presence of outliers. Soares et al. converted the localization problem into a convex optimization problem using simple fast convex relaxation, and then they tested the method with 10% outlier measurements engaged [30].

It may be noted that the method in [28] is costly and has high complexity. The approaches proposed in [29] and [30] only considered outlier measurements that yielded Gaussian distribution, which was the ideal situation. In this paper, motivated by the above issues, we propose a practical, robust non-cooperative localization algorithm (RNLA) that regards the buoys as sensor nodes and uses RSSI in the presence of outlier measurements in OSNs. Firstly, the localization problem is formulated using RSSI with a log-normal shadowing model and the first-order Taylor series exploited. After considering the dynamics of sensor nodes, the length of the anchor chain of the buoys, and the depth of the water, the moving area is restricted. Moreover, because of the non-Gaussian outlier measurements involved, the maximum likelihood estimator cannot function well. We then convert the original localization problem into an optimization problem using squared range and weighted least square (WLS), albeit in a nonconvex form. Furthermore, the optimization problem is transformed into a generalized trust region subproblem (GTRS) incorporated with robust functions. Despite that GTRS is still a nonconvex framework, we conduct a bisection-based block prox-linear (BPL) method to solve it. Additionally, a Cramer–Rao low bound (CRLB) is acquired to evaluate the proposed method.

The main differences between this paper and the previous works are: (1) We consider the dynamics of all nodes, which is more practical in real situations, especially in such a highly dynamic ocean environment. (2) We take into account the length of the anchor chain of the buoys and the depth of the sea, restricting the moving area. (3) In some previous works, localization in OSNs contains two procedures (i.e., detecting the outliers and then eliminating the outliers before locating the nodes); however, in this paper, we take the outlier measurements into consideration without detecting and eliminating procedures, directly locating the unknown nodes in the presence of outlier measurements, which may save localization time in OSNs. The major contributions of this paper can be concluded as these two aspects: (1) we convert the localization problem into an optimization framework combined with robust functions, and (2) a robust algorithm named RNLA, which figures out the global solution and works well in a highly dynamic ocean environment, is proposed for localization in OSNs.

The remainder of the paper is organized as follows. In Section 2, we introduce the problem formulation of non-cooperative localization. In Section 3, the proposed algorithm is illustrated. In Section 4, comprehensive simulation results are discussed. In the last section, Section 5, we conclude this paper.

## 2. Problem Formulation

**Figure 1:**
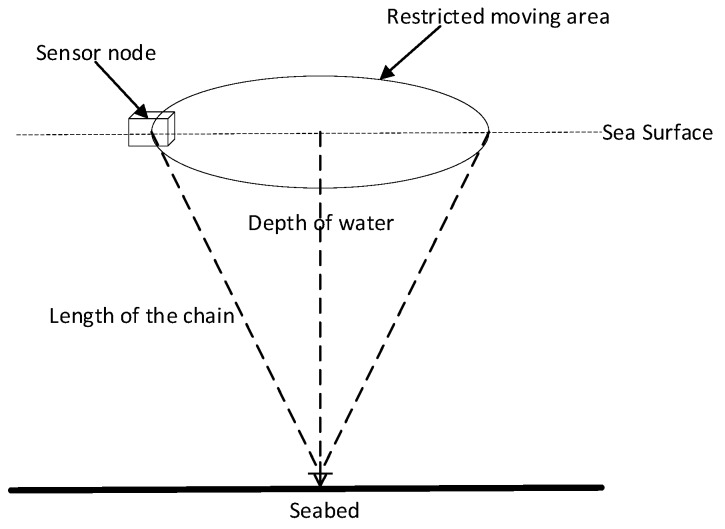
Restricted area for moving sensor nodes.

Assume plenty of sensor nodes (buoys with anchor chain attached) are deployed on the ocean surface of interest. In this paper, we assumed all sensor nodes were movable in the restricted area, which is the base of the cone shown in Figure 1. Without loss of generality, suppose the number of anchor nodes and unknown nodes are N and M respectively. The position of anchor nodes and unknown nodes are $A^{t}={\left[{\left(a_{1}^{t}\right)}^{T},{\left(a_{2}^{t}\right)}^{T},\ldots ,{\left(a_{N}^{t}\right)}^{T}\right]}^{T}$ and $U^{t}={\left[{\left(u_{1}^{t}\right)}^{T},{\left(u_{2}^{t}\right)}^{T},\ldots ,{\left(u_{M}^{t}\right)}^{T}\right]}^{T}$ at time t. T presents the transpose. For the $i^{th}$ anchor node and the $j^{th}$ unknown node at time t, the positions can be expressed as $a_{i}^{t}=\left[a_{ix}^{t},a_{iy}^{t}\right],i\in N$ and $u_{j}^{t}=\left[u_{jx}^{t},u_{jy}^{t}\right],j\in M$ respectively. x and y indicate the corresponding coordinate.

In this paper, we employed RSSI as the ranging method because its time does not need to be synchronized, it is cost-effective, and it saves energy [9]. The localization procedures were executed in a centralized manner, where all information within the communication radius collected was transmitted to the processing center. The tradeoff information at each time slot between anchor nodes and unknown nodes included the RSSI value and corresponding identity number. Suppose all sensor nodes are armed with the signal receiver, which means the nodes are aware of the signal strength. The received RSSI value could be modeled as the propagation path-loss model [10]

(1) $$RSSI_{ri}^{t}=RSSI_{sj}^{t}-PL(d_{0})-10\vartheta \mathrm{lg}\left(\frac{d_{ij}^{t}}{d_{0}}\right)+\eta_{ij}^{t},$$

where $RSSI_{ri}^{t}$ denotes the signal strength received by the $i^{th}$ anchor node from the $j^{th}$ unknown node at time t. $RSSI_{sj}^{t}$ denotes the transmission power of the $j^{th}$ unknown node at time t. $PL(d_{0})$ is the signal strength loss value when the reference distance is $d_{0}=1$ m. ϑ indicates the path loss factor. $d_{ij}^{t}$ denotes the distance between the $i^{th}$ anchor node and the $j^{th}$ unknown node at time t. $\eta_{ij}^{t}$ is Gaussian white noise, which is rewritten as η for the convenience in the rest of the paper.

Assume R is the matrix that contains all range measurements, $RSSI_{ri}^{t}=P_{ri}^{t}$, $RSSI_{sj}^{t}-PL(d_{0})=P_{sj}^{t}$, and $d_{0}=1$ m. If the noise follows a zero-mean Gaussian distribution with variance σ, the corresponding probability density function (PDF) can be written as Equation (2) [10].

(2) $$p(R^{t}|U^{t})=\prod_{j=1}^{M}\prod_{i=1}^{N}\frac{1}{\sqrt{2\pi \sigma^{2}}}\exp\left\{-\frac{{\left\{P_{ri}^{t}-P_{sj}^{t}+10\vartheta \mathrm{lg}(d_{ij}^{t})\right\}}^{2}}{2\sigma^{2}}\right\}.$$

If the transmission power and the path loss factor are known, Formula (1) could be rewritten as Equation (3) [10].

(3) $${\left(d_{ij}^{t}\right)}^{2}=10^{\frac{P_{sj}^{t}-P_{ri}^{t}}{5\vartheta }}\cdot 10^{\frac{\eta }{5\vartheta }}.$$

When the noise is relatively small, the right side of Equation (3) can be approximated using the first-order Taylor series expansion as Equation (4) [20].

(4) $$10^{\frac{P_{sj}^{t}-P_{ri}^{t}}{5\vartheta }}\cdot 10^{\frac{\eta }{5\vartheta }}\approx 10^{\frac{P_{sj}^{t}-P_{ri}^{t}}{5\vartheta }}\cdot (1+\frac{\ln10}{5\vartheta }\eta ).$$

Equation (4) can be, alternatively, written as Equation (5) [20]

(5) $$10^{\frac{P_{sj}^{t}-P_{ri}^{t}}{5\vartheta }}\cdot 10^{\frac{\eta }{5\vartheta }}\approx 10^{\frac{P_{sj}^{t}-P_{ri}^{t}}{5\vartheta }}+\eta^{\prime },$$

where $\eta^{\prime }=10^{\frac{P_{sj}^{t}-P_{ri}^{t}}{5\vartheta }}\cdot \frac{\ln10}{5\vartheta }\eta$.

Now, the problem in Equation (2) can be reformulated as Equation (6) [20].

(6) $$p(R^{t}|U^{t})=\prod_{j=1}^{M}\prod_{i=1}^{N}\frac{1}{\sqrt{2\pi \sigma^{2}}}\exp\left\{-\frac{{\left(\left\|a_{i}^{t}-u_{j}^{t}\right\|-d_{ij}^{t}\right)}^{2}}{2\sigma^{2}}\right\}.$$

Notably, Equation (6) could be solved by the maximum likelihood (ML) estimator. However, once the outlier measurements exist (the non-Gaussian noise attributes to the noise distribution), the ML estimator, to some extent, malfunctions. Henceforth, the proposed RNLA method was motivated by this case.

In this paper, we took into account a non-Gaussian noise situation. The noise distribution consisted of two parts: (1) Gaussian distribution and (2) uncertain distribution (the distribution of outlier measurements) [31].

(7) $$p(\eta )=(1-\beta )\Omega \left(\eta ;0,\sigma^{2}\right)+\beta \varphi (\eta ),$$

where $\Omega \left(\eta ;0,\sigma^{2}\right)$ denotes the Gaussian distribution. φ(η) indicates the outlier measurements distribution. β is the contamination ratio.

## 3. Proposed Algorithm

### 3.1. Generalized Trust Region Subproblem (GTRS)

In Section 2, the ML estimator was conducted when the noise was Gaussian. However, the ML estimator was not functional in the presence of non-Gaussian outlier measurements. Therefore, we converted Equation (6) into the optimization problem using squared range and WLS.

(8) $$\hat{U}=\arg\min{\sum_{j=1}^{M}\sum_{i=1}^{N}\omega_{i}^{t}\left\{{\left\|a_{i}^{t}-u_{j}^{t}\right\|}_{2}^{2}-{\left(d_{ij}^{t}\right)}^{2}\right\}}^{2},$$

where $\omega_{i}^{t}$ denotes the weight at time t.

It should be noted that the problem in Equation (8) is nonconvex. Thus, a proper transformation is exploited by reformulating it as a constrained minimization problem [32]. Assume that each unknown node is independent. Here, we took one of the unknown nodes j as an example to demonstrate the rest of the parts. Then, Equation (8) can be rewritten as

(9) $$\begin{array}{l} ({\hat{u}}_{j}^{t},{ℑ}_{j}^{t})=\arg\min\sum_{i=1}^{N}\omega_{i}^{t}{\left({ℑ}_{j}^{t}-2{\left(a_{i}^{t}\right)}^{T}u_{j}^{t}+{\left\|a_{i}^{t}\right\|}^{2}-{\left(d_{ij}^{t}\right)}^{2}\right)}^{2}\\ \;\;\;\;\;\;\mathrm{subject}\;\mathrm{to}\;{\left\|u_{j}^{t}\right\|}^{2}={ℑ}_{j}^{t} \end{array}.$$

Now, the problem in Equation (9) can be expressed as a quadratic program as

(10) $$\begin{array}{l} \underset{y_{j}^{t}}{\mathrm{minimize}}\;{\left\|\omega^{t}({℘}^{t}y_{j}^{t}-b^{t})\right\|}^{2}\\ \mathrm{subject}\;\mathrm{to}\;(y_{j}^{t}{)}^{T}Dy_{j}^{t}+2f^{T}y_{j}^{t}=0 \end{array},$$

where $y_{j}^{t}={\left[u_{j}^{t},{ℑ}_{j}^{t}\right]}^{T}$ and the corresponding matrices ${℘}^{t}$, $b^{t}$, D, and f are defined as

(11) $${℘}^{t}=\left[\begin{array}{cc} \begin{array}{l} -2{\left(a_{1}^{t}\right)}^{T}\\ \vdots \\ -2{\left(a_{N}^{t}\right)}^{T} \end{array} & \begin{array}{l} 1\\ \vdots \\ 1 \end{array} \end{array}\right],b^{t}=\left[\begin{array}{l} {\left(d_{ij}^{t}\right)}^{2}-{\left\|a_{1}^{t}\right\|}^{2}\\ \vdots \\ {\left(d_{Nj}^{t}\right)}^{2}-{\left\|a_{N}^{t}\right\|}^{2} \end{array}\right],D=\left[\begin{array}{cc} \begin{array}{l} I_{n}\\ 0_{1\times n} \end{array} & \begin{array}{l} 0_{n\times 1}\\ 0 \end{array} \end{array}\right],f=\left[\begin{array}{l} 0_{n\times 1}\\ -\frac{1}{2} \end{array}\right],$$

where n denotes the dimension.

The original localization problem is further transformed into GTRS, a constrained minimization problem, as shown in Equation (10).

### 3.2. German-McClure (GM) Function with Huber Norm in GTRS

Although the problem has already been transformed into GTRS, large errors may appear in a way, directly using a bisection approach to figure out the solution under outlier measurements. Henceforth, inspired by [33,34], the objective function could be expressed as

(12) $$J\left(y_{j}^{t},\omega^{t}\right)=\sum_{i=1}^{N}\omega_{i}^{t}{\left({℘}_{i}^{t}y_{j}^{t}-b_{i}^{t}\right)}^{2}+\sum_{i=1}^{N}\varepsilon^{2}\omega_{i}^{t}-\ln\omega_{i}^{t},$$

where ${℘}_{i}^{t}=[-2{\left(a_{i}^{t}\right)}^{T},1]$, $b_{i}^{t}={\left(d_{ij}^{t}\right)}^{2}-{\left\|a_{i}^{t}\right\|}^{2}$, and ε denotes the parameter that needs to be determined.

The former summation of Equation (12) is Equation (10), the latter term of Equation (12) is added to cater for the use of the German-McClure (GM) function, being one of the M-estimators commonly used in robust statistics. The aim of the GM function is to degrade the influence of large errors by interpolating between $\ell_{2}$ and $\ell_{0}$ norm [35].

The GM function can be expressed as

(13) $$\rho_{\varepsilon }(e)=\frac{e^{2}}{e^{2}+\varepsilon^{2}},$$

where e denotes the residual of the former summation of Equation (12). $\rho_{\varepsilon }(\cdot )$ indicates the function of e.

However, the GM function does not guarantee a unique solution, though it is a robust function. In this case, the Huber norm, being a convex function, is incorporated with the GM function to figure out parameter e.

The Huber norm is introduced in Equation (14).

(14) $$\rho_{\kappa }(e)=\left\{ \begin{array}{ll} \frac{1}{2}e^{2} & \left|e\right|<\kappa \\ \kappa \left|e\right|-\frac{\kappa^{2}}{2} & \left|e\right|\geq \kappa \end{array}\right..$$

If the noise is Gaussian, the Huber function would be 95% asymptotically efficient when the parameter κ is 1.34σ [34].

Incorporating the GM function with the Huber norm, Equation (14) can be rewritten as

(15) $$\rho_{\varepsilon }(e)=\left\{ \begin{array}{ll} \frac{e^{2}}{e^{2}+\varepsilon^{2}} & \left|e\right|<\varepsilon_{0}\\ \frac{1}{8}(\frac{3}{\varepsilon_{0}}\left|e\right|-1) & \left|e\right|\geq \varepsilon_{0} \end{array}\right.,$$

where $\varepsilon_{0}=1.34\sigma$ and $\varepsilon =\sqrt{3}\varepsilon_{0}$.

Now, we have obtained the optimal parameter $\varepsilon =1.34\sqrt{3}\sigma$.

### 3.3. Robust, Non-Cooperative Localization Algorithm (RNLA)

The problem in Equation (12) can be expressed as

(16) $$\begin{array}{l} \underset{y_{j}}{\mathrm{minimize}}{\left({℘}^{t}y_{j}^{t}-b^{t}\right)}^{T}W_{k-1}^{t}({℘}^{t}y_{j}^{t}-b^{t})\\ \;\;\;\mathrm{subject}\;\mathrm{to}\;(y_{j}^{t}{)}^{T}Dy_{j}^{t}+2f^{T}y_{j}^{t}=0 \end{array},$$

where $W_{k}^{t}$ denotes the diagonal weight matrix in the $k^{th}$ iteration at time t, wherein ${\left(\omega_{i}^{t}\right)}^{k}$ is the $i^{th}$ diagonal entry of $W_{k}^{t}$.

At each iteration, the value of ${\left(\omega_{i}^{t}\right)}^{k}$ is updated by ${\left(y_{j}^{t}\right)}^{k}$ and ${\left(\omega_{i}^{t}\right)}^{k-1}$. The value of ${\left(y_{j}^{t}\right)}^{k+1}$ will be updated by ${\left(y_{j}^{t}\right)}^{k}$ and ${\left(\omega_{i}^{t}\right)}^{k}$. Hence, the weight of the $k^{th}$ iteration of $i^{th}$ diagonal entry at time t is given by

(17) $${\left(\omega_{i}^{t}\right)}^{k}=\frac{1}{{\left\{{\left(e_{i}^{t}\right)}^{k}\right\}}^{2}+\varepsilon^{2}},$$

where ${\left(e_{i}^{t}\right)}^{k}={℘}_{i}^{t}{\left(y_{j}^{t}\right)}^{k}-b_{i}^{t}$.

It is worth noting that the GTRS has no duality gap, and the optimal solution can be derived from the dual solution [32]. ${\left(y_{j}^{t}\right)}^{k}$ would be the optimal solution of Equation (16) within a necessary and sufficient condition:

(18) $$\begin{array}{l} \left\{{\left({℘}^{t}\right)}^{T}W_{k-1}^{t}{℘}^{t}+\lambda^{t}D\right\}{\left(y_{j}^{t}\right)}^{k}={\left({℘}^{t}\right)}^{T}W_{k-1}^{t}b^{t}-\lambda^{t}f,\\ {\left\{{\left(y_{j}^{t}\right)}^{k}\right\}}^{T}D{\left(y_{j}^{t}\right)}^{k}+2f^{T}{\left(y_{j}^{t}\right)}^{k}=0,\\ {\left({℘}^{t}\right)}^{T}W_{k-1}^{t}{℘}^{t}+\lambda^{t}D\underline{≻}0. \end{array}$$

The last term of Equation (18) means that ${\left({℘}^{t}\right)}^{T}W_{k-1}^{t}{℘}^{t}+\lambda^{t}D$ is a positive semidefinite. Under the constraint conditions, the problem of Equation (16) is reformulated to figure out the optimal solution of $\lambda^{t}$.

(19) $$\phi (\lambda^{t})={\left\{{\left(y_{j}^{t}\right)}^{k}\left(\lambda^{t}\right)\right\}}^{T}D{\left(y_{j}^{t}\right)}^{k}\left(\lambda^{t}\right)+2f^{T}{\left(y_{j}^{t}\right)}^{k}\left(\lambda^{t}\right);$$

(20) $${\left(y_{j}^{t}\right)}^{k}\left(\lambda^{t}\right)={\left\{{\left({℘}^{t}\right)}^{T}{℘}^{t}+\lambda^{t}D\right\}}^{-1}\left\{{\left({℘}^{t}\right)}^{T}b^{t}-\lambda^{t}f\right\}.$$

To ensure ${\left({℘}^{t}\right)}^{T}W_{k-1}^{t}{℘}^{t}+\lambda^{t}D\underline{≻}0$, the optimal of $\lambda^{t}$ denoted $\lambda^{t1*}$ should be in the interval ξ [32]

(21) $$\lambda^{t1*}\geq \xi =-\frac{1}{\lambda_{1}},$$

where $\lambda_{1}$ is the largest eigenvalue of ${\left\{{\left({℘}^{t}\right)}^{T}{℘}^{t}\right\}}^{-\frac{1}{2}}D{\left\{{\left({℘}^{t}\right)}^{T}{℘}^{t}\right\}}^{-\frac{1}{2}}$.

In pursuance of ${\left(y_{j}^{t}\right)}^{k}$, we should figure out $\lambda^{t*}$ first, which is solved by a bisection approach.

However, only the theoretical convergence is guaranteed from a bisection approach. For the sake of the global convergence of the solution, we exploit BPL [36] in the bisection procedure.

Suppose the estimate of the $j^{th}$ unknown node in the $k^{th}$ iteration at time t is ${\left(y_{j}^{t}\right)}^{k}$, and the corresponding weight is $W_{{}^{k}}^{t}$. Inspired by [36], the update rule for $y_{j}^{t}$ can be expressed as

(22) $$\begin{array}{l} {\left(y_{j}^{t}\right)}^{k}=\underset{y_{j}^{t}}{\arg\min}\langle \nabla_{y_{j}^{t}}J\left({\left({\hat{y}}_{j}^{t}\right)}^{k},W_{k-1}^{t}\right),y_{j}^{t}-{\left({\hat{y}}_{j}^{t}\right)}^{k}\rangle +l_{k}^{t}{\left\|y_{j}^{t}-{\left({\hat{y}}_{j}^{t}\right)}^{k}\right\|}_{2}^{2}\\ \;\;\;\;\;\;\mathrm{subject}\;\mathrm{to}\;(y_{j}^{t}{)}^{T}Dy_{j}^{t}+2f^{T}y_{j}^{t}=0 \end{array}$$

where $l_{k}^{t}$ is the Lipschitz constant of $\nabla_{y_{j}^{t}}J\left(y_{j}^{t},W_{k-1}^{t}\right)$.

(23) $$\upsilon_{k}^{t}=\frac{1}{12}\sqrt{\frac{l_{k-1}^{t}}{l_{k}^{t}}};$$

(24) $${\left({\hat{y}}_{j}^{t}\right)}^{k}={\left(y_{j}^{t}\right)}^{k}+\upsilon_{k+1}^{t}\left({\left(y_{j}^{t}\right)}^{k-1}-{\left(y_{j}^{t}\right)}^{k-2}\right).$$

In general, BPL is a variant of the gradient descent method. Here we use ${\left(y_{j}^{t}\right)}^{k}$ as the initial value to figure out the optimal solution $y_{j}^{t^{*}}$ under the update rule and extrapolation factor $\upsilon_{k}^{t}$ [36]. ${\left(y_{j}^{t}\right)}^{k+e}$ is the optimal solution $y_{j}^{t^{*}}$ when the conditions below are satisfied:

(25) $$\left(l_{k+e}^{t}I_{n+1}+\lambda^{t}D\right){\left(y_{j}^{t}\right)}^{k+e}=-{\left({℘}^{t}\right)}^{T}\upsilon_{k+e-1}^{t}\left({℘}^{t}{\left({\hat{y}}_{j}^{t}\right)}^{k+e}-b^{t}\right)+l_{k+e}^{t}{\left({\hat{y}}_{j}^{t}\right)}^{k+e}-\lambda^{t}f;$$

(26) $$\begin{array}{l} {\left\{{\left(y_{j}^{t}\right)}^{k+e}\right\}}^{T}D{\left(y_{j}^{t}\right)}^{k+e}+2f^{T}{\left(y_{j}^{t}\right)}^{k+e}=0,\\ \lambda^{t2*}\geq \max\left\{-l_{k+e}^{t},-\frac{1}{\lambda_{1}}\right\}. \end{array}$$

It is apparent that the problem in Equation (26) is GTRS, of which $\lambda^{t2*}$ is obtained through iteration processes. Once $\lambda^{t2*}$ is acquired, the corresponding ${\left(y_{j}^{t}\right)}^{k+e}$ could be obtained by the first term of Equation (25).

The proposed RNLA method could be expressed in Algorithm for RNLA. (Algorithm 1)
**Algorithm 1.** Algorithm for RNLA1. Initiation: A, ε, maximum number of iterations $Iteration=Iteration^{1}+Iteration^{2}$, the convergence tolerance δ and ς, and time2. Calculate the range measurement matrix R3. **for**
t=1:time
**do**4.  **for**
j=1:M
**do**5.   **for**
i=1:N
**do**6.    Calculate ${℘}^{t}$, $b^{t}$, D, and f according to Equation (11)7.   **end**8.   Let ${\left(\omega_{i}^{t}\right)}^{0}=1,\forall i$ and k=19.   **while** STOP = FALSE **do**10.    **Solve** Equation (19) with Equation (20) using the bisection approach to figure out $\lambda^{t1*}$.11.    **Update**
${\left(y_{j}^{t}\right)}^{k}$ according to Equation (20)12.    **Update**
${\left(\omega_{i}^{t}\right)}^{k}$ according to Equation (17)13.     **if**
$\left|J\left({\left(y_{j}^{t}\right)}^{k},W_{k}^{t}\right)-J\left({\left(y_{j}^{t}\right)}^{k-1},W_{k-1}^{t}\right)\right|<\delta$ or reach the number of $Iteration^{1}$
**then**14.       STOP←True15.     **else**
k=k+116.   **end**17.   Let $l_{k}^{t}=0$, $\upsilon_{k}^{t}=0$18.   **while** STOP = FALSE **do**19.      **Calculate**
$l_{k}^{t}=2{\left\|{\left({℘}^{t}\right)}^{T}W_{k}^{t}{℘}^{t}\right\|}_{F}$20.      **Calculate**
$\upsilon_{k}^{t}$ and ${\left({\hat{y}}_{j}^{t}\right)}^{k}$ according to Equations (23) and (24)21.      **Figure out**
$\lambda^{t2*}$ according to Equation (26)22.      **Update**
${\left(y_{j}^{t}\right)}^{k}$ according to Equation (25)23.      **Update**
${\left(\omega_{i}^{t}\right)}^{k}$ according to Equation (17)24.       **if**
$\left|{\left(y_{j}^{t}\right)}^{k}-{\left(y_{j}^{t}\right)}^{k-1}\right|<\varsigma$ or reach the number of Iteration
**then**25.          STOP←True26. **      else**
k=k+127.     **end**28.   **end**29. **end**

**Figure 2:**
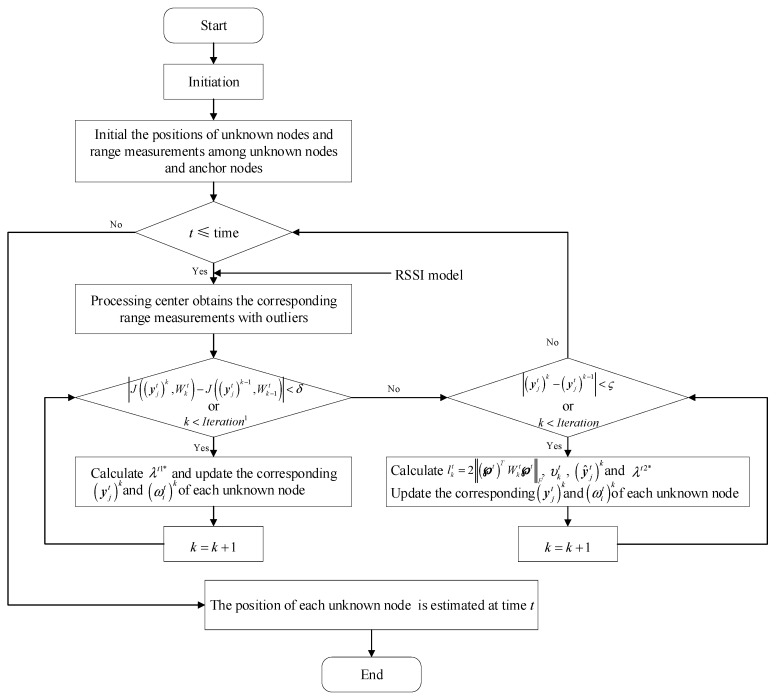
Flowchart of the robust, non-cooperative localization algorithm (RNLA). RSSI = received signal strength indication.

In addition, a flowchart is presented, as shown in Figure 2, to better understand the process of RNLA.

### 3.4. Cramer–Rao Low Bound (CRLB)

In this part we will conduct the CRLB, being a covariance matrix representing a lower bound of any unbiased estimators [37], for the location estimate. Same as the above parts, we take the $j^{th}$ unknown node at time t as an example. Let ${\hat{u}}_{j}^{t}=\left[{\hat{u}}_{jx}^{t},{\hat{u}}_{jy}^{t}\right]$ denote the estimated position of the $j^{th}$ unknown node at time t, and $Cov\left({\hat{u}}_{j}^{t}\right)$ denote the covariance matrix. Basically, the covariance will meet

(27) $$Cov\left({\hat{u}}_{j}^{t}\right)=E\left\{{\left({\hat{u}}_{j}^{t}-u_{j}^{t}\right)}^{T}\left({\hat{u}}_{j}^{t}-u_{j}^{t}\right)\right\}\underline{≻}F^{-1},$$

where F is the Fisher information matrix (FIM), which can be expressed as

(28) $$F=\left[\frac{\partial^{2}p\left(R^{t}|u_{j}^{t}\right)}{\partial^{2}u_{j}^{t}}\right].$$

Only two parameters are included in $u_{j}^{t}$, thus Equation (28) could be rewritten as

(29) $$F=\left[\begin{array}{cc} \frac{\partial^{2}p\left(R^{t}|u_{j}^{t}\right)}{\partial^{2}u_{jx}^{t}} & \frac{\partial p\left(R^{t}|u_{j}^{t}\right)}{\partial u_{jx}^{t}}\cdot \frac{\partial p\left(R^{t}|u_{j}^{t}\right)}{\partial u_{jy}^{t}}\\ \frac{\partial p\left(R^{t}|u_{j}^{t}\right)}{\partial u_{jy}^{t}}\cdot \frac{\partial p\left(R^{t}|u_{j}^{t}\right)}{\partial u_{jx}^{t}} & \frac{\partial^{2}p\left(R^{t}|u_{j}^{t}\right)}{\partial^{2}u_{jy}^{t}} \end{array}\right].$$

It is easy to obtain closed-form expressions of F if the noise yields Gaussian distribution. However, in this paper, the noise is not Gaussian distribution due to outlier measurements are engaged. The closed-form expressions are not available. In this matter, a Monte Carlo simulation is employed. Equation (29) can be rewritten as

(30) $$F={\left(\frac{10\vartheta }{\ln10}\right)}^{2}\cdot I_{\eta }\cdot \left[\begin{array}{cc} \sum_{i=1}^{N}\frac{\left(u_{jx}^{t}-a_{ix}^{t}\right)\left(u_{jx}^{t}-a_{ix}^{t}\right)}{{\left\|u_{j}^{t}-a_{i}^{t}\right\|}_{2}^{4}} & \sum_{i=1}^{N}\frac{\left(u_{jx}^{t}-a_{ix}^{t}\right)\left(u_{jy}^{t}-a_{iy}^{t}\right)}{{\left\|u_{j}^{t}-a_{i}^{t}\right\|}_{2}^{4}}\\ \sum_{i=1}^{N}\frac{\left(u_{jy}^{t}-a_{iy}^{t}\right)\left(u_{jx}^{t}-a_{ix}^{t}\right)}{{\left\|u_{j}^{t}-a_{i}^{t}\right\|}_{2}^{4}} & \sum_{i=1}^{N}\frac{\left(u_{jy}^{t}-a_{iy}^{t}\right)\left(u_{jy}^{t}-a_{iy}^{t}\right)}{{\left\|u_{j}^{t}-a_{i}^{t}\right\|}_{2}^{4}} \end{array}\right],$$

where $I_{\eta }\approx \frac{1}{N_{C}}\sum_{sample=1}^{N_{C}}\frac{{\left[\nabla_{\eta }p{\left(\eta \right)}^{sample}\right]}^{2}}{p^{2}\left({\left(\eta \right)}^{sample}\right)}$ denotes the intrinsic error that can be obtained by Monte Carlo simulation. $N_{C}$ denotes the total number of samples in the Monte Carlo simulation. $\nabla_{\eta }p\left(\eta \right)$ is the first gradient operator.

Let ${\left\|{\hat{u}}_{j}^{t}-u_{j}^{t}\right\|}_{2}=error$. The root mean square error (RMSE) is related to the obtained CRLB through

(31) $$\sqrt{E\left(error^{2}\right)}\geq \sqrt{Tr\left(F^{-1}\right)}≜CRLB\left(u_{j}^{t}\right),$$

where Tr(⋅) is the trace of a matrix.

### 3.5. Complexity Analysis

The complexity of the algorithm is strongly relative to N and M, specifically, the function of N and M. Assuming that the network is fully connected, the number of connections in the network could be expressed as C=M⋅N+M⋅(M−1)/2 [38]. At each time slot, the unknown nodes will be located at a time. Since we utilized a non-cooperative scheme, there were no connections among unknown nodes. In this case, if the network was fully connected, the number of connections of the network should be C=M⋅N. For an unknown node (i.e., M=1), let *Iteration* be the maximum number of steps. At each time slot, the corresponding complexity of RNLA could be linear (i.e., O(Iteration⋅N)). In other words, the overall complexity of the network could be O(Iteration⋅M⋅N).

## 4. Numerical Simulations

In this section, several experiments were carried out in different scenarios in Matlab R2018b to verify the effectiveness of RNLA. The simulation area was 500 × 500 m^2^, wherein the depth of water was 40 m, and the length of the anchor chain was 50 m. Basically, if data were collected for the wind speed and the level of the wave, the corresponding mobility model for sensor nodes could be acquired after analyzing the interactive force exerted on the nodes. Hence, due to absence of the data, we assumed all nodes were movable in a random walk model in the restricted area with a velocity of 2 m/s. We set the number of outlier measurements as N×β varying from the anchor nodes. The number of Monte Carlo simulations was 100, where $N_{C}=1000$. The distribution of outlier measurements could be a uniform distribution, an exponential distribution, a Rayleigh distribution, etc. Here, in this paper, we assumed the distribution of outlier measurements was a uniform distribution (considering it was convenient) in which the assumption was commonly exploited in many works [39,40,41,42]. For example, the authors in [40] constructed a deep regression model with the use of a Gaussian-uniform mixture model to deal with the outliers. The authors in [41] modeled the distribution of the measurement as a Gaussian-uniform mixture in order to handle the severe performance degradation under interference. In this paper, the uniform distribution follows $\left(-500\sqrt{2},500\sqrt{2}\right)$. CRLB and three methods—the method Directly Squaring Least Square (DS-LS) mentioned in [11], the method Weighted Least Square-Known parameters (WLS-K) proposed in [20], and the method called Weighted Triangle Centroid Algorithm (WTCA) proposed in [43]—as comparisons were involved. The reason that we chose these three methods was that they were proposed for localization using RSSI in OSNs. In addition, DS-LS and WLS-K converted the localization problem into an optimization framework, which was one of the contributions of the paper, though the optimization functions were different. Moreover, WLS-K basically could be recognized as the common solution for GTRS, wherein we combined a bisection approach and the BPL method to solve GTRS in this paper. Although WTCA did not convert the localization problem into an optimization framework, this method was from our previous work that considered the high dynamics of the ocean environment. In this paper, we calibrated localization accuracy by RMSE, which can be expressed as

(32) $$RMSE=\sqrt{\frac{1}{M\cdot time}\sum_{t=1}^{time}\sum_{j=1}^{M}{\left(u_{jx}^{t}-{\hat{u}}_{jx}^{t}\right)}^{2}+{\left(u_{jy}^{t}-{\hat{u}}_{jy}^{t}\right)}^{2}}.$$

### 4.1. Scenario with Variable Anchor Nodes

In this part, the simulation was executed under variable anchor nodes. The corresponding parameters were as follows: M = 100, σ = 2 m, and β = 0.4.

**Figure 3:**
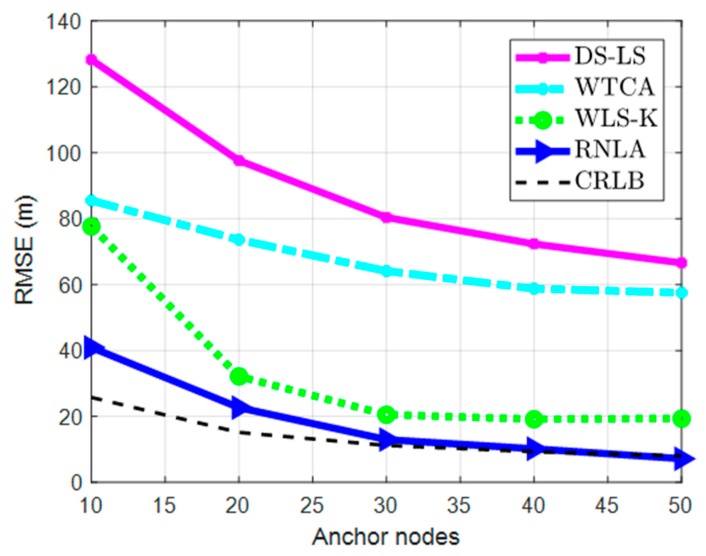
Root mean square error (RMSE) under variable anchor nodes. CRLB = Cramer–Rao low bound. Directly Squaring Least Square = DS-LS. Weighted Triangle Centroid Algorithm = WTCA. Weighted Least Square-Known parameters = WLS-K.

The performances of different methods under variable anchor nodes are indicated in Figure 3. As expected, the performance of the methods improved by increasing the number of anchor nodes. This was because more measurement information was provided for unknown nodes as the number of anchor nodes grew. However, because there were outlier measurements, the performances of the methods were inconsistent. DS-LS was affected mostly because a direct squaring strategy was utilized in the method, in which the performance depended on relative, accurate measurements. As for WTCA, the method was a microelectromechanical system (MEMS)-aided algorithm, where the accumulated error grew as time passed. Thus, the performance was worse than WLS-K, wherein an iterative reweighed method was used, which alleviated the influence of outlier measurements on localization (in a way). Nevertheless, because robust functions and the BPL method were used, the negative influence of outlier measurements received by the unknown nodes reduced gradually at each iteration. Hence, RNLA outperformed the other three methods and was close to CRLB.

### 4.2. Scenario with Variable β

In this scenario, the contamination ratio β was changeable. Parameters were set as: N = 20, M = 100, and σ = 2 m. 

**Figure 4:**
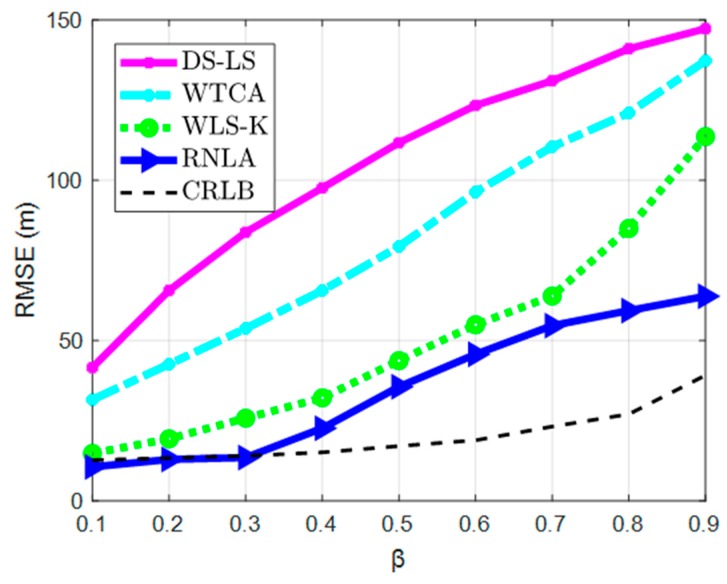
RMSE under variable β.

In Figure 4, we can see that the performances of all methods deteriorated during an increase of β, or equivalently, the rise of outlier measurements. The performance of DS-LS was the worst in comparison because outlier measurements influenced the ranging. The same situation occurred in WTCA, though the performance was better than DS-LS. It should be noted that, because of the accumulated error resulting from MEMS, the deterioration ratio of WTCA was larger than DS-LS. The ratio of WLS-K augmented over the increase of β as well. When the value of β was small, close performances were exhibited between WLS-K and RNLA. This was because the iterative reweighed strategy worked at a few outlier measurements. However, the difference became more distinguishable as β increased. The optimal parameter conducted by robust functions worked at each iteration when β increased. Hence, though localization accuracy was getting worse, the deterioration ratio of RNLA decreased significantly, and the performance was better than others.

### 4.3. Scenario with Variable σ

In this part, simulations were carried out under variable σ with or without outlier measurements. In addition, to verify the effectiveness of the robust functions, WLS-K-Robust was conducted in the simulations. Parameters were set as: N = 20, M = 100, and β = 0 or β = 0.4.

**Figure 5:**
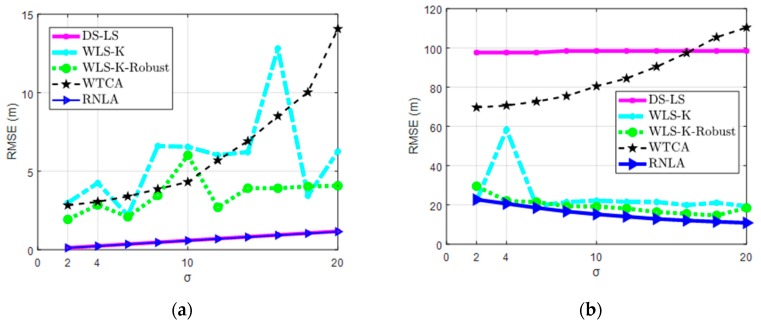
RMSE under variable σ: (**a**) Localization performance without outlier measurements, and (**b**) Localization performance with outlier measurements.

According to Equation (7), if β = 0, the second term of Equation (7) is equal to zero, meaning that there are no outlier measurements. Hence, the scenario in Figure 5a indicates the localization without outlier measurements. Another one, Figure 5b, is the scenario of localization with outlier measurements.

In Figure 5, the performance of WTCA was significantly worse than others while σ increased if the outlier measurements engaged or not. On the contrary, DS-LS performed well, close to RNLA, and outperformed WLS-K, WLS-K-Robust, and WTCA (Figure 5a), although it performed badly in the presence of outlier measurements shown in Figure 5b. Regarding WLS-K, whether the robust functions engaged had a divergent performance. In Figure 5a,b, WLS-K-Robust worked more steadily or even better than WLS-K. Besides, we knew that the robust functions could alleviate the adverse effect of σ on localization from Figure 5b. Over and above the robust functions, RNLA integrated with BPL, which further improved performance and outperformed the others in the presence or absence of outlier measurements.

### 4.4. Cumulative Distribution Function (CDF)

**Figure 6:**
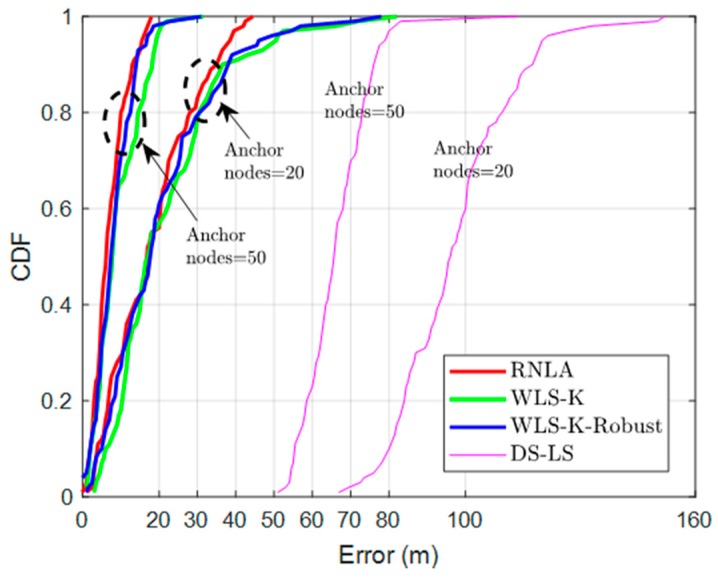
Cummulative distribution function (CDF) of different methods.

In order to demonstrate the effectiveness of the proposed method further, we conducted the simulation of CDF shown in Figure 6. Resulting from the increase of accumulated error by MEMS, it was unfair to make a comparison for WTCA. Accordingly, only WLS-K, WLS-K-Robust, and DS-LS were involved in this part.

Despite the performance of DS-LS, which was strongly sensitive to outlier measurements and the worst one in Figure 6, we focused more on the other three methods. Figure 6 shows that RNLA beat WLS-K and WLS-K-Robust, improving the localization accuracy by more than 12 m and 35 m when N = 50 and N = 20, respectively, on average. We can also see that RNLA achieved error < 20 m and error < 50 m at 100% when N = 50 and N = 20, respectively, whereas others attained the same probability in the case of error ≥ 30 m and error ≥ 70 m respectively.

### 4.5. Computation Time

**Figure 7:**
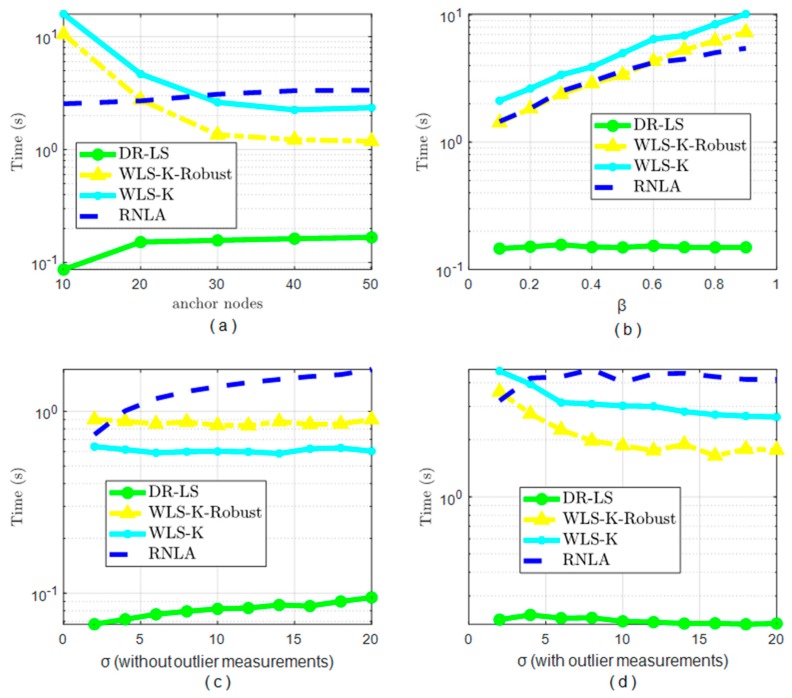
The computation times of different methods under different conditions: (**a**) Computation times under variable anchor nodes, (**b**) Computation times under variable β, (**c**) Computation times under variable σ without outlier measurements, and (**d**) Computation times under variable σ with outlier measurements.

Computation time, or equivalently computation efficiency, was another factor to calibrate the performance of the methods, apart from the localization accuracy. Unfortunately, RNLA was not flawless. In Figure 7, the computation time in some situations was not satisfactory, though it had an outstanding localization accuracy with or without outlier measurements as shown in Figure 3 to Figure 6. It should be noted that WTCA was a MEMS-aided algorithm, wherein only one-time localization was carried out. After one-time localization, acquiring the location was the task of MEMS. Thus, the scheme was almost a real-time one. This was the reason we did not compare WTCA computation times in Figure 7.

The maximum number of iterations of RNLA that we set in the simulation was 1000. In Figure 7, we saw that the robust functions could improve the computation efficiency, besides improving the localization accuracy, in the presence of outlier measurements. DR-LS was the most efficient one in all scenarios, whereas RNLA was the worst one, on average. This was because the BPL, a method to figure out the global solution, involved RNLA, which needed extra time for searching. However, the computation time of RNLA was less than WLS-K and WLS-K-Robust when N≤20 (in the first scenario) and less than WLS-K, close to WLS-K-Robust, in the second scenario (variable β). Hence, if the area of interest did not have enough anchor nodes engaged in localization, expecting to have a good performance in the presence of outlier measurements simultaneously, RNLA seemed to be the better one to locate the unknown nodes. But if the environment was ideal (no outlier measurements), of course, there was no doubt that DR-LS was the best choice. Although the computational efficiency was not satisfactory in comparing DR-LS, WLS-K, and WLS-K-Robust, the average time for computation was 2.4 s, which was far less than our previous cooperative work in [10].

## 5. Conclusions

In this paper, a practical localization method named RNLA using RSSI in the presence of outlier measurements in OSNs was proposed. The localization problem was formulated firstly using a log-normal shadowing model and the first-order Taylor series. However, there were non-Gaussian outlier measurements, which the maximum likelihood estimator could not function well with. Thus, we then converted the original localization problem into an optimization problem using squared range and WLS, albeit in a nonconvex form. Furthermore, integrated robust functions with the optimization problem, a GTRS framework, was conducted. To obtain the global optimal solution, BPL was incorporated with a bisection procedure. In addition, CRLB was acquired to evaluate the proposed method. Several experiments were executed under variable parameters and compared to other methods. Because the robust functions and the BPL method (a method for searching the global solution) were engaged in the algorithm, the negative influence of outlier measurements on localization gradually reduced after iteration. Therefore, RNLA outperformed the others, though the computation time was a little bit longer than some of them in some scenarios. In future work, we will verify the proposed method in a real situation, considering more dynamic scenarios. In addition, methods with less computation times will be investigated in OSNs in the future.
